# Genetic contributions of noncognitive skills to academic development

**DOI:** 10.21203/rs.3.rs-2775994/v1

**Published:** 2023-04-07

**Authors:** Margherita Malanchini, Andrea G. Allegrini, Michel G. Nivard, Pietro Biroli, Kaili Rimfeld, Rosa Cheesman, Sophie von Stumm, Perline A. Demange, Elsje van Bergen, Andrew D. Grotzinger, Laurel Raffington, Javier De la Fuente, Jean-Baptiste Pingault, K. Paige Harden, Elliot M. Tucker-Drob, Robert Plomin

**Affiliations:** 1 School of Biological and Behavioural Sciences, Queen Mary University of London, United Kingdom; 2 Social, Genetic and Developmental Psychiatry Centre, King’s College London, United Kingdom; 3 Department of Clinical, Educational and Health Psychology, University College London, United Kingdom; 4 Department of Biological Psychology, Vrije Universiteit Amsterdam, Amsterdam, the Netherlands; 5 Department of Economics, Universita’ di Bologna, Bologna, Italy; 6 Royal Holloway University of London, United Kingdom; 7 PROMENTA Research Center, Department of Psychology, University of Oslo, Oslo, Norway; 8 Department of Education, University of York, United Kingdom; 9 Research Institute LEARN!, Vrije Universiteit Amsterdam, Amsterdam, the Netherlands; 10 Amsterdam Public Health Research Institute, Mental Health, Amsterdam, the Netherlands; 11 Institute for Behavioral Genetics, University of Colorado Boulder, United States; 12 Max Planck Research Group Biosocial – Biology, Social Disparities, and Development; Max Planck Institute for Human Development, Berlin, Germany; 13 Department of Psychology, The University of Texas at Austin, United States

## Abstract

Noncognitive skills such as motivation and self-regulation, predict academic achievement beyond cognitive skills. However, the role of genetic and environmental factors and of their interplay in these developmental associations remains unclear. We provide a comprehensive account of how cognitive and noncognitive skills contribute to academic achievement from ages 7 to 16 in a sample of >10,000 children from England and Wales. Results indicated that noncognitive skills become increasingly predictive of academic achievement across development. Triangulating genetic methods, including twin analyses and polygenic scores (PGS), we found that the contribution of noncognitive genetics to academic achievement becomes stronger over development. The PGS for noncognitive skills predicted academic achievement developmentally, with prediction nearly doubling by age 16, pointing to gene-environment correlation (rGE). Within-family analyses indicated both passive and active/evocative rGE processes driven by noncognitive genetics. By studying genetic effects through a developmental lens, we provide novel insights into the role of noncognitive skills in academic development.

## Introduction

Children who are emotionally stable, motivated, and capable of regulating their attention and impulses do better in school, independent of their level of cognitive ability^1–7^. These important socioemotional characteristics have been broadly described as *noncognitive skills*^8^. “Noncognitive” is an imperfect term that primarily serves to differentiate these characteristics from what they are not – performance on standardized tests of cognitive ability. The panoply of noncognitive skills that predicts better educational outcomes includes a wide range of psychological characteristics that can be organized into three partly overlapping domains: motivational factors, self-regulatory strategies and personality traits^9^.

Twin research has shown that genetic differences between people contribute to their differences in noncognitive skills. This is reflected in the finding that most domains of noncognitive skills, including academic motivation^10,11^, self-regulation^12^ and personality^13^ are moderately heritable (~30–50%). In addition, twin studies have found evidence that noncognitive skills are genetically correlated with academic achievement^14,15^. That is, some of the same genetic differences that are associated with variation in academic achievement are also associated with noncognitive skills.

DNA-based methods confirmed genetic links between noncognitive skills and academic performance. A series of genome-wide association studies (GWAS) of educational attainment (*i.e*., years of formal education completed) has identified genetic variants that are correlated with completing formal education^16,17^. A polygenic score (PGS) constructed from these GWASs results predicts higher levels of self-control^18^, more adaptive personality traits (higher conscientiousness, agreeableness, and openness to experience), and greater academic motivation^19^. A new method, called GWAS-by-subtraction^20^, has allowed to model associations between DNA variants and educational attainment that were independent of cognitive test performance to obtain a GWAS of noncognitive skills. The genetics of noncognitive skills were found to be related to conscientiousness, openness to experience, delay of gratification, and health-risk behaviours. These in-depth characterizations have provided us with the first insight into the genetics of educationally relevant noncognitive skills.

**Figure 1.**
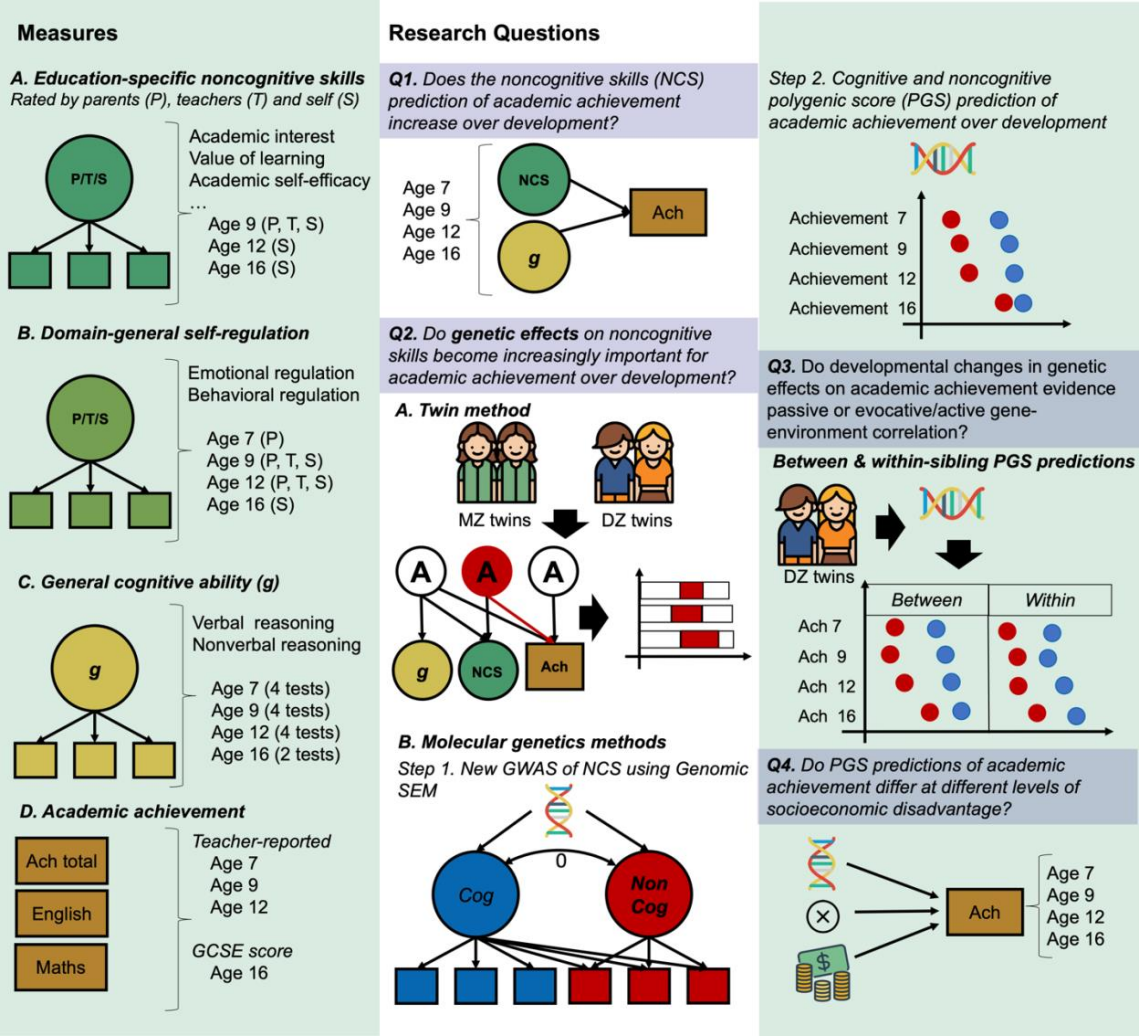
A visual summary of the measures, research questions and methods adopted in the present study. **Left panel:** We used factor analysis to capture individual differences in two broad dimensions of noncognitive skills: education-specific noncognitive skills (including measures such as academic interest, academic self-efficacy and value attributed to learning), and domain-general self-regulation skills (including measures of behavioural and emotional regulation not necessarily related to the school context). We also created latent measures of general cognitive ability from verbal and nonverbal cognitive tests at ages. Academic achievement measures included teacher ratings of academic performance based on the national curriculum at ages 7, 9 and 12 and exam scores at age 16 (see Methods for a detailed description). **Center and right panels:** A summary of the methodologies adopted to address each of the four core research questions in the study. We addressed the first research question (Q1) by conducting a series of multiple regressions to investigate changes in the developmental contribution of noncognitive skills to academic achievement beyond cognitive skills. We addressed the second research question (Q2) using multiple genetic methods. First (A), we conducted trivariate Cholesky decompositions using twin data. Second (B), we created a new GWAS of noncognitive skills by extending the GWAS-by-subtraction (Demange et al., 2021) approach with a set of GWAS for specific cognitive tasks and SES-relevant traits and examined developmental changes in the cognitive (Cog) and noncognitive (NonCog) polygenic score prediction of academic achievement from age 7 to 16. We addressed our third research question (Q3) by modelling Cog and NonCog PGS effects within a sibling difference design, therefore separating within-family from between-family effects. We investigated our fourth research question (Q4) fitting multivariable models including the effects of the Cog/NonCog PGS, family socioeconomic status, and their two-way interaction.

The current study aims to address four important questions regarding the role of noncognitive skills in academic development that remain unanswered (Figure 1). First, does the association between noncognitive skills and academic achievement change developmentally? Research has highlighted how skills that are broadly considered noncognitive, such as for example self-control, rely heavily on cognitive competencies^21^, therefore it is important to consider the role of cognitive skills when assessing the relationship between noncognitive skills and academic achievement. Developmental studies that have examined the contribution of cognitive and noncognitive skills in conjunction remain scarce and focused on a few specific measures and relatively short time frames^22^. Here we take a systematic approach to investigate the association between noncognitive skills and academic achievement throughout compulsory education, accounting for cognitive skills.

Second, do genetic dispositions towards noncognitive skills become increasingly important for academic achievement across development? Twin studies focusing on specific moments in childhood^23^ or adolescence^24^, have found that noncognitive skills such as motivation and self-regulation contribute to academic achievement and its heritability beyond cognitive skills^25^. However, to our knowledge no study to date has examined this question systematically by adopting a developmental framework. We leverage a comprehensive battery of developmental data on cognitive and noncognitive skills collected from over 10,000 children born in England and Wales (Figure 1, left panel) to investigate the developmental stability and change in the contribution of genetic factors associated with cognitive and noncognitive skills to academic achievement. We triangulated evidence across multiple methods, including twin and PGS analyses.

Evidence of an increasingly important role of genetic effects on academic achievement over development is consistent with a transactional model of human development^26^, rooted in gene-environment correlation (rGE), the idea that environmental exposure is partly dependent on genetic disposition^27,28^. Three types of rGE have been proposed: passive, evocative and active^26,27^. Passive rGE refers to how, by virtue of growing up with their biological relatives, children experience environments that correlate with their genotype. Parental genotypes are likely to impact offspring’s phenotypes via both genetic and environmental pathways, as parents shape the family environment partly depending on their own genetic dispositions. In addition, children might evoke or actively seek environmental experiences that correlate with their genetic dispositions^27^. For example, children with a greater genetic disposition towards academic achievement might find learning easier and might therefore evoke positive reactions from their educators that, in turn, might result in greater achievement. Children with a greater disposition towards academic achievement might also actively select, modify, and create environmental experiences that are in line with such disposition, for example by deciding to take advanced classes. Through rGE, genetic differences between children can therefore result in differential exposure to learning environments, which, in turn, can affect their academic achievement. As a result of these processes, genetic similarity would result in an increased phenotypic similarity and consequently in an increase in genetic effects through environmental processes^29,30^.

Third, to what extent developmental changes in genetic effects on academic achievement evidence passive or evocative/active rGE? One way of disentangling the effects of different types of rGE is to model polygenic score effects within a sibling difference design^31^. Within-sibling analyses rely on how the transmission of alleles from parents to offspring is randomized during meiosis, such that siblings have an equal probability of inheriting any given allele, independently of environmental processes. Therefore, genetic differences between siblings are thought to be free from demographic confounding factors, since these operate at the population level, not at the within-family level, and free from environmental influences shared by the siblings, which include passive rGE. Evidence of increasing genetic prediction over development at the within-family level would therefore be free from the effects of demography and passive rGE, and be consistent with the possibility of active and/or evocative rGE^32^. Therefore, in a third set of analyses we investigate to what extent developmental changes in the polygenic score prediction of academic achievement are due to between versus within-family processes.

Fourth, do genetic effects on academic development depend on environmental context? Genetic and environmental processes might interact such that the effects of environmental experiences on a trait might be partly dependent on genetic effects^33,34^. Studies that have examined this possibility have focused on the role of socioeconomic disadvantage across a broad range of contexts, including family socioeconomic status^35,36^ and the school environment^37,38^. In a last set of analyses, we explore whether the cognitive and noncognitive PGS prediction of academic achievement differs at different levels of socioeconomic disadvantage across development. Adopting a multi-method, developmental approach, these analyses (see preregistration here: https://osf.io/m5f7j/) address four core research questions providing a detailed account of the processes through which cognitive and noncognitive skills are linked to the development of individual differences in academic achievement.

## Results

### Noncognitive skills predict academic achievement beyond cognitive skills with increasingly strong effects across development.

Different measures of noncognitive skills were available at different ages, and these were provided by different raters: parents, teachers, and self-rated by the twins. Based on extant literature and measures availability, we focused on two broad dimensions of noncognitive skills: 1) education-specific noncognitive skills, including measures such as academic interest, attitudes towards learning, and academic self-efficacy 2) domain-general self-regulation skills, such as measures of behavioural and emotional regulation not necessarily related to the school context (Figure 1 and Methods). Here we report analyses conducted on these two latent dimensions, and we report the outcomes of the analyses for individual noncognitive measures in the Supplementary Material (Supplementary Note 1, Supplementary Figure 1, and Supplementary Tables 1 and 2).

Latent factors of education-specific noncognitive skills and domain-general self-regulation skills constructed using factor analysis (Supplementary Tables 3 and 4 and figure 1) correlated positively with academic achievement at all developmental stages. Effect sizes differed depending on the rater and developmental stage considered but tended to increase with age. For example, the association between self-rated education-specific noncognitive skills and academic achievement increased from small (*r* = 0.10) at age 9, to moderate (*r* = 0.41) at age 12, to strong (*r* = 0.51) at age 16 (see Supplementary Note1, Supplementary Figure 2 and Supplementary Table 5). Latent noncognitive factors were also modestly correlated with latent factors of general cognitive ability (Supplementary Table 6) at the same age (Supplementary table 7).

**Figure 2.**
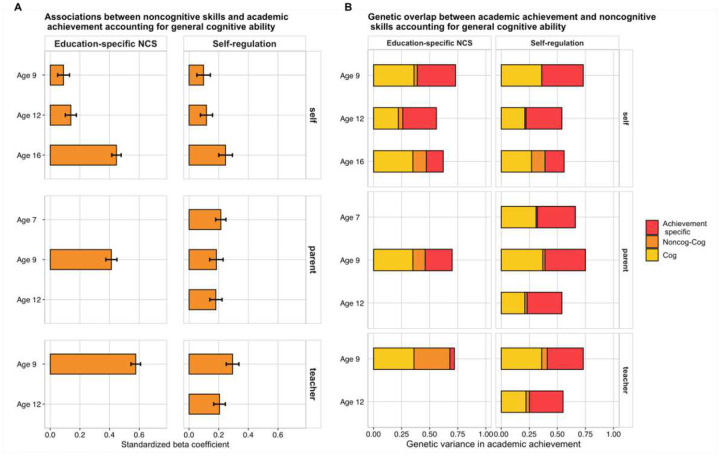
**(Panel A).** Associations between latent factors of noncognitive skills and academic achievement at ages 7, 9, 12 and 16, after accounting for general cognitive ability at the same age using multiple regression. Each bar indicates the effect size of standardized regression coefficients and the error bars indicate the 95% confidence intervals around the estimates. The left panel shows the associations for latent measures of education-specific noncognitive skills (NCS), while the right panel the associations for latent dimensions of domain-general self-regulation skills. The figure is further divided into self-rated (top panel), parent-rated (middle panel) and teacher-rated (bottom panel) measures. **(Panel B).** Each bar represents genetic effects on academic achievement over development and includes three shadings. The lighter (yellow) shadings indicate the proportion of genetic variance in academic achievement that can be attributed to genetic variance in cognitive skills (Cog). The orange shadings indicate the proportion of genetic variance in academic achievement that can be attributed to genetic variance in noncognitive skills, independent of the genetics of cognitive skills (Noncog – Cog). The red shadings indicate genetic effects on academic achievement independent of the genetics of cognitive and noncognitive skills (Achievement specific). Results are further divided into self-rated (top panel), parent-rated (middle panel) and teacher-rated (bottom panel) measures. 95% Confidence intervals for all estimates are presented in Supplementary Tables 12 and 13.

We examined whether general cognitive ability could account for the associations between noncognitive skills and academic achievement. Results of multiple regression analyses showed that both noncognitive factors were substantially and significantly associated with academic achievement beyond cognitive skills at every stage of compulsory education (Figure 2A and Supplementary Table 8). The relative contribution of noncognitive skills to academic achievement increased developmentally, particularly when considering self-reported measures. For self-reported education-specific noncognitive skills, the effect size of the relative prediction of achievement increased from *β* = .10 at age 9 (when the effect size for the cognitive prediction was *β* = .46) to *β* = .28 at age 12 (when the cognitive prediction was *β* = .36) to *β* = .58 at age 16 (when the cognitive prediction was *β* = .39). A developmental increase was also observed for self-reported measures of domain-general self-regulation skills, for which the predictive power increased from *β* = .11 at age 9 to *β*= .21 at age 16 after accounting for general cognitive ability (Supplementary Table 8).

### Specific genetic associations between noncognitive skills and academic achievement persist after accounting for cognitive skills and increase in magnitude across development.

Applying twin designs (Methods), we found that the heritability (i.e., the extent to which observed differences in a trait are accounted for by genetic differences) of noncognitive skills differed significantly across raters and developmental stages (Supplementary Note 2, Supplementary Table 9, and Supplementary Figures 3–7). The heritabilities of latent noncognitive dimensions ranged between 74% for self-reported domain-general self-regulation skills at age 9 and 93% for parent-reported education-related noncognitive skills at age 9 (Supplementary Note 2, Supplementary Tables 10–11 and Supplementary Figure 8). For these latent dimensions, the correlation between noncognitive measures and academic achievement was mostly accounted for by genetic factors and, to a lower extent, by nonshared environmental factors (Supplementary note 2 and Supplementary Figure 8).

We then investigated whether the observed genetic associations between latent dimensions of noncognitive skills and academic achievement could be accounted for by genetic factors associated with cognitive skills. We investigated this question with a series of trivariate Cholesky decompositions (Methods) the results of which are presented in Figure 2B. Each bar in Figure 2B is the outcome of a different trivariate Cholesky decomposition examining the extent to which genetic effects associated with noncognitive skills accounted for genetic effects on academic achievement after controlling for genetic effects associated with cognitive skills at the same age. The length of each bar indicates the proportion of variance in academic achievement that is accounted for by genetic factors (i.e., heritability). The yellow shadings show that genetic effects associated with cognitive skills account for between 21% and 36% of the total variance in academic achievement. The orange shadings indicate that genetic effects associated with noncognitive skills account for between 0.1% and 32.5% of the variance in academic achievement, independent of cognitive skills. The red shadings show that between 5% and 37% of the variance of academic achievement is independent of genetic effects associated with cognitive and noncognitive skills.

The top panels of Figure 2B illustrate the developmental increase in how the genetics of self-reported noncognitive skills contribute to the genetics of academic achievement. Standardized squared path estimates increased from 1% of the total variance in academic achievement at age 9 to 4% at age 12 and 12% at age 16 (Supplementary Tables 12 and 13). This increase in contribution beyond cognitive skills was observed for both education-specific noncognitive skills and domain-general self-regulation skills (see Supplementary Figure 9 for the full models’ results which include shared and nonshared environmental estimates).

### A more fine-grained genomic measure of cognitive and noncognitive skills.

**Figure 3.**
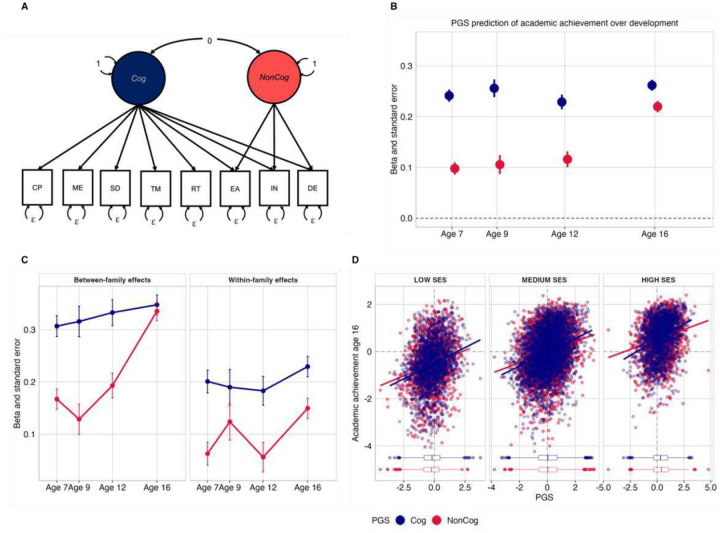
Contribution of noncognitive genetics to academic development: genomic analyses and gene-environment interplay. (Panel A.) Path diagram for the extension of the GWAS-by-subtraction model implemented in genomic structural equation model. In addition, GWAS summary statistics for cognitive performance (CP) and educational attainment (EA), summary statistics of memory (ME), symbol digit (SD), trail making (TM), and reaction time (RT) GWASs loaded on the cognitive (Cog) latent factor while GWAS summary statistics for income (IN) and deprivation (DE) loaded on the noncognitive (NonCog) latent factor, in addition to EA (Methods). (Panel B.) Cognitive and noncognitive polygenic score (PGS) prediction of academic achievement at ages 7,9, 12 and 16. (Panel C.) Results of polygenic scores analyses after partitioning the effects of Cog and NonCog into between and within family factors. (Panel D.) Cognitive (Cog) and noncognitive (Noncog) PGS prediction of academic achievement at the end of compulsory education (age 16), plotted at different levels of family socioeconomic status (SES).

We investigated whether we could observe a developmental increase in the contribution of noncognitive genetics to academic achievement using genomic methods. To better understand what was captured by the cognitive and noncognitive genetic factors we extended the GWAS-by-subtraction model^20^. On the one hand, we extended the latent cognitive factor by including more nuanced cognitive measures (episodic memory; processing speed, executive functions, and reaction time^39^ GWAS summary statistics). On the other hand, we were particularly interested in whether the noncognitive factor captured something separate from socioeconomic status. Therefore, we extended the non-cognitive factor to explicitly model socioeconomic variables (including Townsend Deprivation and Income^40^) in addition to educational attainment^17^. This noncognitive factor was therefore defined as genetic variation shared by education and socioeconomic status that was independent of all measured cognitive abilities. Akin to Demange et al. 2021, we then fitted a Cholesky model (Methods) where indicators of the noncognitive latent factor (henceforth NonCog) were regressed on the cognitive latent factor (henceforth Cog; Figure 3A and Supplementary Table 14).

The newly created cognitive and noncognitive factors correlated strongly with those obtained from Demange et al.^20^ (Supplementary Table 15). The genetic correlation was 0.96 for the cognitive factors and 0.93 for the noncognitive factors. The genetic correlation between Cog and NonCog was rg = 0.15. Supplementary Figure 10 shows the genetic correlations between the newly created Cog and NonCog genetics and 18 psychiatric, personality and socio-economic traits, which we compared to the genetic correlations obtained by Demange et al.^20^. The pattern of associations was largely consistent across the two models. However, in some instances, results diverged. Specifically, with respect to psychiatric traits, autism, anorexia, and ADHD, a larger gap was observed between the cognitive and noncognitive factors, as compared to Demange et al., where differences in the correlations were less pronounced or absent. As expected, the results differed most for socioeconomic traits, with stronger correlations for NonCog than Cog with longevity (r = 0.52 Vs. r = 0.34), neighbourhood deprivation (r = −0.66, Vs. r = −0.28), and educational attainment (r = 0.83 Vs. r = 0.65; Supplementary Figure 10 and Supplementary Table 15).

### The noncognitive polygenic prediction of academic achievement increases over development.

In our independent sample, we calculated PGS for Cog and NonCog (Methods) and examined their prediction of cognitive, noncognitive and academic traits over development. We first investigated whether and to what extent Cog and NonCog PGS predicted individual differences in noncognitive skills across development by modelling both PGSs in a multiple regression model (Methods). In line with our previously obtained results showing a moderate association between cognitive and noncognitive traits, we found that the Cog PGS significantly predicted variation in noncognitive skills across development, with standardized effect sizes ranging between ß = 0.04 and ß = 0.22 (Figure S11 and Supplementary Table 16). The NonCog PGS, independent of the cognitive PGS, predicted observed variation in noncognitive skills at all developmental stages. Associations were small at earlier ages (e.g., ß = 0.07, SE = 0.02, p(corrected) = 1.93E-03) for parent-reported education-specific noncognitive skills at 9, and ß = 0.10, SE = 0.01, p(corrected) = 2.24E-11 for parent-reported self-regulation at 7) but they increased developmentally, particularly for self-reported education-specific noncognitive measures (ß = 0.16, SE = 0.02, p(corrected) = 8.30E-17 at age 16). The only exception was observed for self-reported education-specific noncognitive skills at age 9, for which the prediction was negative (ß = −0.03, SE = 0.02) and did not reach significance after accounting for multiple testing (Supplementary Table 16).

Cog and NonCog PGSs predicted variation in general cognitive ability, verbal ability, and nonverbal ability at all developmental stages. As expected, the Cog PGS prediction of cognitive phenotypes was substantially stronger than the NonCog prediction, with estimates ranging between ß = 0.19 and ß = 0.27 for the Cog PGS and between ß = 0.10, p(corrected) = 4.41E-10 and ß = 0.18, p(corrected) 5.51E-21 for the NonCog PGS (Supplementary Table 16).

Next, we considered the effects of the Cog and NonCog PGSs on academic achievement over development. We detected associations between the Cog PGS and achievement as early as age 7 (ß =0.24, SE = 0.01, p(corrected) = 3.68E-86), these associations remained largely consistent across development (ß = 0.26, se = 0.01, p(corrected) = 2.71E-126 at age 16). Although we observed weaker effects of the NonCog PGS in early childhood (ß =0.10, SE = 0.01, p(corrected) = 8.12E-15) as compared to the Cog PGS, these increased across development and reached effects comparable to those of the Cog PGS at age 16 (ß =0.22, SE = 0.01, p(corrected) = 1.85E-84; Figure 3B and Supplementary Table 16). The same pattern of associations was observed also when considering achievement in English and mathematics, separately (Supplementary Table 16). This observed increase in the NonCog PGS prediction of academic achievement over development is consistent with transactional models of gene-environment correlation, driven by noncognitive genetics. These PGS predictions were in line with those obtained using the PGSs created using the GWAS-by-subtraction method published by Demange et al. (Supplementary Table 17).

### Within-family polygenic score analyses indicate both passive and evocative/active gene-environment correlation supported by noncognitive processes

Given our findings of an increase in the NonCog polygenic contributions to academic achievement across development, consistent with the possibility of transactional processes of gene-environment correlation (rGE), we extended our preregistered analyses to delve deeper into these putative rGE effects. We investigated whether the pattern of associations found across development could be explained by passive or evocative/active rGE, with a family-level fixed-effect regression. Specifically, we separated within-family effects (also indexing direct genetic effects) from between-family (population-level) effects, also capturing the effects of passive rGE and demography, by modelling polygenic score effects for cognitive and noncognitive skills within a sibling difference design (Methods). We examined the within and between siblings Cog and NonCog PGS prediction of academic achievement from age 7 to 16.

Two main findings emerged from this analysis. First, we observed that the effect sizes for the direct effects of NonCog were about half if compared to population-level associations (Supplementary Table 18). Similarly, the prediction from the Cog PGS was reduced by over one-third, consistent with previous evidence^31^. Second, while the Cog direct and indirect genetic effects did not change substantially over development (range ß = 0.20 to ß = 0.23), NonCog effects increased steeply developmentally (range ß = 0.06 to ß = 0.15; Figure 3C, and Supplementary Table 18). This pattern of results could be observed for both direct and indirect genetic effects. This suggested that the developmental increase in PGS prediction was mostly driven by noncognitive rather than cognitive skills. In addition, the developmental increase observed for both indirect and direct effects suggested that both passive and evocative/active rGE processes might be at play. The increase in the noncognitive PGS prediction at the between-family (indirect effects) level is consistent with the possibility of passive gene-environment correlation processes. The increase in the noncognitive PGS effects at the within-family level (direct effects) is consistent with evocative/active rGE because the effects of passive rGE are removed. We conducted sensitivity analyses, and replicated the results, with the PGSs constructed using the method published by Demange et al. (Supplementary Table 18b).

### Does socioeconomic status modify the association between Cog/NonCog PGS and educational outcomes across development?

Lastly, we extended our preregistered analyses to test whether socio-economic status (SES) could explain or modify the observed pattern of developmental associations between PGS and academic achievement. We fitted multivariable models at each developmental stage including Cog/NonCog PGS effects, along with SES at recruitment, covariates, and their two-way interactions (see Methods) to test whether SES moderated Cog and NonCog PGS effects on academic achievement. After adjusting for SES, the same pattern of relationships was observed, with a relatively stable association between the Cog PGS and achievement, and a steeper increase in the NonCog PGS prediction, even though all effects were attenuated (Supplementary Table 19). We did not detect significant interaction effects between either the Cog or the NonCog PGS with SES (Supplementary Table 19).

Figure 3D depicts mutually adjusted slopes for the Cog and NonCog PGS prediction against academic achievement at different levels of family SES. The figure shows that although higher SES corresponded to greater achievement on average, the slope of the association between the Cog and NonCog PGS and achievement did not differ across socio-economic strata. Higher PGS, for both cognitive and noncognitive skills, corresponded to higher academic achievement, and higher SES corresponded to both higher mean PGSs and higher achievement, indicating a correlation rather than an interaction between genetic and environmental influences on academic achievement.

## Discussion

We investigated the contribution of cognitive and noncognitive genetics to the development of individual differences in academic achievement during compulsory education in a UK-based sample. Four complementary findings emerged: First, the noncognitive skills prediction of academic achievement increases over the school years, and these effects remain substantial after accounting for cognitive skills. Second, the contribution of noncognitive skills to academic achievement is mainly due to common genetic factors, whose influence increases over the school years. For example, the noncognitive polygenic score prediction of academic achievement nearly doubles over the school years, while the cognitive polygenic score prediction remains relatively stable. Third, this increasingly important role of noncognitive genetics persists even after accounting for family-fixed effects. Fourth, polygenic score contributions to academic development did not differ across socio-economic contexts. Together, these findings highlight the important role that noncognitive skills play during primary and secondary education, emphasize how these skills contribute to academic development through a complex process of interplay between genetic and environmental factors, and suggest that fostering such skills might provide an avenue for successful educational strategies and interventions.

The first set of novel findings about development emerged from twin analyses of the covariance between noncognitive traits and academic achievement. First, we found that genetic factors accounted for most of the observed correlations between noncognitive skills and academic achievement at all developmental stages. Second, both phenotypic and genetic correlations increased developmentally, particularly for self-reported measures of noncognitive traits. Third, our twin analyses showed that genetic factors accounted for most of the correlations between noncognitive skills and academic achievement after accounting for cognitive skills. Finally, this independent genetic contribution of noncognitive skills to academic achievement increased developmentally. This increase was observed for both education-specific noncognitive skills, where the measures included in the general factors changed developmentally, as well as for domain-general self-regulation skills, for which the same measures were collected at all developmental stages. Therefore, the observed developmental increase in phenotypic and genetic associations independent of cognitive skills is unlikely to be an artefact of inconsistencies in measurement, but it likely reflects the increasingly important role of noncognitive skills across compulsory education.

A further aim of the current study was to better understand what was captured by the noncognitive PGS constructed using GWAS-by-subtraction^20^, particularly in relation to what other skills beyond cognitive ability propel students down different educational trajectories. Given the link between socioeconomic status and academic achievement^41^ we were specifically interested in whether the noncognitive PGS indexed something separate from socio-economic-related factors. To this end, we extended the GWAS-by-subtraction model in two directions. First, with the aim of making a more refined cognitive factor, we added summary statistics from several other GWASs of fluid intelligence. Second, we included GWASs of other traits known to associate with achievement beyond cognitive abilities, specifically targeting SES-related traits such as income and social deprivation, making the noncognitive PGS factor more explicitly socio-economic relevant. Interestingly, the results obtained from this new model paralleled those we obtained when we applied the cognitive and noncognitive PGSs from the original GWAS-by-subtraction model, suggesting that the PGS measure of noncognitive skills from Demange et al. already captured some SES-related effects.

Paralleling our multivariate twin results, we observed that the effects of the prediction from noncognitive PGS to academic achievement increased from childhood to adolescence, beyond effects of the cognitive PGS. A few explanations are possible for this finding. First, this could be attributable to gene-environment correlation (rGE), which could be passive, evocative or active^27,42^. Another explanation could be that PGS become increasingly predictive during development because the sample becomes closer in age to the adult samples where GWAS effect sizes were estimated in the case of educational attainment and cognitive performance^17^. However, it is of note that this increase in prediction was not observed for the cognitive PGS, for which effects on academic achievement were mostly stable developmentally, suggesting that this relationship might be a consequence of transactional processes of gene-environment correlation that contribute to academic development via noncognitive genetics.

To explore the role of evocative and active gene-environment correlation in these associations free from the role of passive gene-environment correlation and demography, we applied a within-sibling design^31^ to test whether family fixed effects could explain the observed increase in the predictive power of the noncognitive PGS. While the contributions of both PGSs were attenuated within-family, suggesting a substantial role for shared family processes (e.g. SES), an increase in the contribution of noncognitive PGS to academic achievement from age 7 to 16 was still evident. In contrast, the contribution of the cognitive PGS remained relatively stable. The increase in the noncognitive PGS prediction at the between-family level is consistent with passive gene-environment correlation processes, while the increase in the noncognitive PGS prediction at the within-family level is consistent with transactional processes driven by active or evocative gene-environment correlation^26,29,42^ for noncognitive PGS. As children grow up, they actively evoke or shape their environmental experiences based in part on their genetic dispositions, and these experiences in turn contribute to their academic development. Our findings suggest that, children’s educational experiences are increasingly shaped by their propensity towards noncognitive skills.

To delve deeper into the role of socio-economic factors, we tested whether SES could modify the relationship between cognitive and noncognitive PGSs and academic achievement over development. While we did not find evidence for interaction effects in this regard, it is of note that cognitive and noncognitive PGS were conditionally independent in a multivariable model including SES, suggesting that the polygenic component captured by the noncognitive skills factor was at least partly independent of SES-related genetic and environmental effects. Future work could focus on investigating whether this noncognitive component can be explicitly separated from SES-like factors, for example extending the approach employed herein with a GWAS-by-subtraction of three factors including intelligence, SES and a noncognitive residual independent of the first two factors.

One caveat of these gene-environment interaction analyses is that adjusting for a heritable covariate, SES in this case, can yield biased estimates in multivariable models including PGS^43,44^. Future work is needed to determine whether this is the case, perhaps leveraging results of within-family GWAS to construct PGS for ‘direct’ effects within families^45^. This limitation also pertains to our within-sibling PGS analyses to adjust for family fixed effects and separate direct from indirect contributions of cognitive and noncognitive PGS to achievement. It has been noted that, depending on the setting, it might be difficult to separate direct and indirect effects using population-based GWAS effects as a starting point^46^. Follow-up of these analyses employing PGS for direct effects obtained from family-based GWAS will shed light on this potential limitation. A further caveat of the present work is that, while we investigated genetic effects on noncognitive skills and their link with academic achievement across development, we did not investigate stability and change using longitudinal models. Future work explicitly investigating developmental change at the phenotypic^47^, genetic^48^ and genomic^49,50^ level, for example using latent growth models^51^, will address further developmental questions related to the role of noncognitive skills in academic development.

To conclude, our study provides an in-depth investigation of the role of noncognitive genetics in academic development. Triangulating multiple genetic and genomic methods, we found consistent evidence for the increasingly important role that noncognitive skills play during compulsory education. Genetic dispositions towards noncognitive skills become increasingly predictive of academic achievement and, by late adolescence, they explain as much variance in achievement as do genetic dispositions towards cognitive skills. Within-family analyses highlighted how these developmental trends are consistent with passive rGE as well as transactional processes of gene-environment correlation by which, as they grow up, children evoke and actively select academic environments that correlate with their genetic disposition towards noncognitive skills^27,42^. Therefore, fostering noncognitive skills might provide a successful avenue for educational interventions.

## Methods

### Sample

Participants are part of the Twins Early Development Study (TEDS), a longitudinal study of twins born in England and Wales between 1994 and 1996. The families in TEDS are representative of the British population for their cohort in terms of socio-economic distribution, ethnicity and parental occupation. Ten thousand families are still actively involved with the TEDS study over twenty years after the first data collection wave (see^52^ for additional information on the TEDS sample). The present study includes data collected in TEDS across multiple waves. Specifically, we will analyze data collected over five collection waves, when the twins were 4, 7, 9, 12 and 16 years old. The sample size differs between collection waves, numbers for all measures included in the study are reported in Supplementary Table 1.

### Measures

Below we provide a brief description of all the measures included in the present study. Please refer to https://www.teds.ac.uk/datadictionary for detailed descriptions of each measure and information on the items included in each construct.

#### Education-specific noncognitive skills

At **age 9** data on education-specific noncognitive skills were collected from parents, teachers and self-reports from the twins. Measures of academic self-perceived ability^53^, academic interest^53^ and the Classroom Environment Questionnaire (CEQ^54^) were available from all raters. The CEQ included the following subscales rated by parents and twins: (1) CEQ classroom satisfaction scale; (2) CEQ educational opportunities scale; (3) CEQ adventures scales, assessing enjoyment of learning. Ratings on the CEQ classroom satisfaction scale were also provided by the teachers.

At **age 12** data on education-specific noncognitive skills were collected from parents, teachers, and self-reports. The following measures were collected: academic self-perceived ability^53^, academic interest^53^, the mathematics environment questionnaire^55^ and the literacy environment questionnaire^56^. The questionnaires asked several questions related to literacy and mathematics, including items such as: *Reading is one of my favourite activities; When I read books, I learn a lot*; and *In school, how often do you do maths problems from text books?* all rated on a four-point Likert scale.

At **age 16** education-specific noncognitive skills were assessed via self-reports provided by the twins. The battery of education-specific noncognitive constructs included the following measures:

- The brief academic self-concept scale included 10 items (adapted from^57^), such as: *I like having difficult work to do* and *I am clever*, rated on a 5-point Likert scale.
- School engagement^58^ includes 5 subscales: teacher-student relations; control and relevance of schoolwork; peer support for learning; future aspirations and goals; family support for learning. The school engagement scale includes items such as: *I enjoy talking to the teachers at my school*, *I feel like I have a say about what happens to me at school*, *School is important for achieving my future goals*, and *When I have problems at school, my family/carer(s) are willing to help me*, rated on a 4-point Likert scale.
- Grit was assessed with 8 items from the Short Grit Scale (GRIT-S)^59^ asking the twins to report on their academic perseverance answering questions such as: *Setbacks don’t discourage me*, and *I am a hard worker,* rated on a 5-point Likert scale.
- Academic ambition^60^ was measured with 5 items asking participants to rate statements like the following on a 5-point Likert scale: *I am ambitious* and *achieving something of lasting importance is the highest goal in life*.
- Time spent studying mathematics was assessed with 3 items asking participants how much time every week they spent in: *Regular lessons in mathematics at school, Out-of school-time lessons in mathematics*, and *Study or homework in mathematics by themselves*.
- Mathematics self-efficacy^61^ was measured with 8 items asking students how confident they felt about having to perform different mathematics tasks, for example: *Calculating how many square metres of tiles you need to cover a floor* and *Understanding graphs presented in newspapers*, rated on a 4-point Likert scale
- Mathematics interest^61^ asked participants to respond to 3 questions related to interest in mathematics, including: I *do mathematics because I enjoy it* and *I am interested in the things I learn in mathematics*.
- Curiosity was assessed with 7 items^62^ asking participants to rate statements such as: *When I am actively interested in something, it takes a great deal to interrupt me* and *Everywhere I go, I am looking out for new things or experiences* on a 7-point Likert scale
- Attitudes towards school was measured using the PISA attitudes to school measure^61^ which included 4 items such as: *School has helped give me confidence to make decisions* and *School has taught me things which could be useful in a job* rated on a 4-point Likert scale.

##### Self-regulation

Emotional and behavioral self-regulation was assessed at all ages using the Strengths and Difficulties Questionnaire (SDQ)^63^. Data on domain-general self-regulation skills was collected from parents, teachers and self-reported by the twins. The SDQ includes 5 subscales: hyperactivity, conduct problems, peer problems, emotional problems, and prosocial behaviour. Composite scores for all subscales except prosocial behaviour were reversed so that higher scores indicated higher levels of domain-general self-regulation skills. At age 7, domain-general self-regulation skills were rated by the parents; at age 9 and 12 by the parents, teachers and self-reported by the twins; and at age 16 self-reported by the twins.

##### Cognitive ability

At **age 7** cognitive ability was measured using four cognitive tests that were administered over the telephone by trained research assistants. Two tests assessed verbal cognitive ability: a 13-item Similarity test and 18-item Vocabulary test, both derived from the Wechsler Intelligence Scale for Children (WISC-III)^64^. Nonverbal cognitive ability was measured using two tests: a 9-item Conceptual Groupings Test^65^, and a 21-item WISC Picture Completion Test^64^. Verbal and nonverbal ability composites were created taking the mean of the standardized test scores within each domain. A *g* composite was derived taking the mean of the two standardized verbal and two standardized nonverbal test scores.

At **age 9** cognitive ability was assessed using four cognitive tests that were administered as booklets sent to TEDS families by post. Verbal ability was measured using the first 20 items from WISC-III-PI Words test^66^ and the first 18 items from WISC-III-PI General Knowledge test^66^. Nonverbal ability was assessed using the Shapes test (CAT3 Figure Classification)^67^ and the Puzzle test (CAT3 Figure Analogies)^67^. Verbal and nonverbal ability composites were created taking the mean of the standardized test scores within each domain. A *g* composite was derived taking the mean of the two standardized verbal and two standardized nonverbal test scores.

At **age 12**, cognitive ability was measured using four cognitive tests that were administered online. Verbal ability was measured using the full versions of the verbal ability tests administered at age 9: the full 30 items from WISC-III-PI Words test^66^ and 30 items from WISC-III-PI General Knowledge test^66^. Nonverbal ability was measured with the 24-item Pattern test (derived from the Raven’s Standard Progressive Matrices)^68^ and the 30-item Picture Completion test (WISC-III-UK)^64^. Verbal and nonverbal ability composites were created taking the mean of the standardized test scores within each domain. A *g* composite was derived from the mean of the two standardized verbal and two standardized nonverbal test scores.

At **age 16** cognitive ability was assessed using a composite of one verbal and one nonverbal test administered online. Verbal ability was assessed using an adaptation of the Mill Hill Vocabulary test^69^, Nonverbal ability was measured using an adapted version of the Raven’s Standard Progressive Matrices test^68^. A *g* composite was derived taking the mean of the two standardized tests.

##### Academic achievement

At **age 7** academic achievement was measured with standardized teacher reports and consisted of standardized mean scores of students’ achievements in English and mathematics, in line with the National Curriculum Level. Performance in English was assessed in four domains: speaking, listening, reading, and writing abilities; performance in maths was assessed in three domains: applying mathematics, as well as knowledge about numbers, shapes, space and measures.

At **age 9,** academic achievement was again assessed using teacher reports. The domains assessed were the same for English and mathematics (although on age-appropriate content). In addition, performance in science was assessed considering two key domains: scientific enquiry and knowledge and understanding of life processes, living things and physical processes.

At **age 12**, academic achievement was assessed in the same way as at age 9, with two exceptions. Mathematics added a fourth domain, data handling, and science added a third domain, materials and their properties. These additions were in line with the changes made to the National Curriculum teacher ratings.

At **age 16**, academic achievement was measured using the General Certificate of Secondary Education (GCSE) exam scores. The GCSE is the UK nationwide examination usually taken by 16-year-olds at the end of compulsory secondary education^70^. Twins’ GCSE scores were obtained via mailing examination results forms to the families shortly after completion of the GCSE exams by the twins. For the GCSE, students could choose from a wide range of subjects. In the current analyses the mean score of the three compulsory GCSE subjects: English Language and/or English Literature, mathematics, and a science composite (a mean score of any of the scientific subjects taken, including physics, chemistry, and biology).

##### Family socio-economic status

At first contact, parents of TEDS twins received a questionnaire by post, and were asked to provide information about their educational qualifications, employment, and mothers’ age at first birth. A socioeconomic status composite was created by standardizing these three variables and calculating their mean. The same measures, except for mother’s age at first birth, were used to measure family socioeconomic status at age 7. At age 16, data on socioeconomic status were collected using a web questionnaire, and a total score was calculated from the standardized mean of 5 items: household income, mother’s and father’s highest qualifications, and mother’s and father’s employment status.

##### Genetic data

Two different genotyping platforms were used because genotyping was undertaken in two separate waves, 5 years apart. AffymetrixGeneChip 6.0 SNP arrays were used to genotype 3,665 individuals. Additionally, 8,122 individuals (including 3,607 DZ co-twin samples) were genotyped on Illumina HumanOmniExpressExome-8v1.2 arrays. Genotypes from a total of 10,346 samples (including 3,320 DZ twin pairs and 7,026 unrelated individuals) passed quality control, including 3,057 individuals genotyped on Affymetrix and 7,289 individuals genotyped on Illumina. The final data contained 7,363,646 genotyped or well-imputed SNPs. For additional information on the treatment of these samples see^71^.

#### Analytic strategies

##### Phenotypic analyses: Confirmatory factor analysis, correlations, and regressions

Confirmatory factor analysis (CFA) was employed to create latent dimensions of noncognitive skills and general cognitive ability at all ages. Based on the well-established literature on general cognitive ability (g) and previous work in the TEDS sample^72^, we constructed one factor for g at each developmental stage. Each g factor was created by taking the weighted loadings of two verbal and two nonverbal tests (see Measures and Supplementary Table 6). CFA was also employed to construct dimensions of noncognitive characteristics. Based on previous meta-analytic work on the noncognitive characteristics that matter for educational outcomes^9,73^, we embraced a theoretical distinction between education-specific noncognitive characteristics (e.g., motivations, attitudes and goals) and broader, more de-contextualized measures of self-regulation (e.g., behavioural and emotional regulation), and created separate factors for a) education-specific noncognitive characteristics and b) domain-general self-regulation skills separately for ages and raters, including all the measures available at each age for each rater (see Supplementary Tables 2 and 3 for factor loadings and model fit indices).

We applied phenotypic correlations to examine the associations between noncognitive skills (both observed measures and factors) and general cognitive ability and academic achievement at each age. We applied multiple regressions to explore the associations between noncognitive skills and academic achievement accounting for general cognitive ability. We applied Benjamini-Hochberg correction^74^ to account for multiple testing.

##### Genetic analyses: The twin method

The twin method allows for the decomposition of individual differences in a trait into genetic and environmental sources of variance by capitalizing on the genetic relatedness between monozygotic twins (MZ), who share 100% of their genetic makeup, and dizygotic twins (DZ), who share on average 50% of the genes that differ between individuals. The method is further grounded in the assumption that both types of twins who are raised in the same family share their rearing environments to approximately the same extent^75^. By comparing how similar MZ and DZ twins are for a given trait (intraclass correlations), it is possible to estimate the relative contribution of genetic factors and environments to variation in that trait. Heritability, the amount of variance in a trait that can be attributed to genetic variance (A), can be roughly estimated as double the difference between the MZ and DZ twin intraclass correlations^75^. The ACE model further partitions the variance into shared environment (C), which describes the extent to which twins raised in the same family resemble each other beyond their shared genetic variance, and non-shared environment (E), which describes environmental variance that does not contribute to similarities between twin pairs (and also includes measurement error).

The twin method can be extended to the exploration of the covariance between two or more traits (multivariate genetic analysis). Multivariate genetic analysis allows for the decomposition of the covariance between multiple traits into genetic and environmental sources of variance, by modelling the cross-twin cross-trait covariances. Cross-twin cross-trait covariances describe the association between two variables, with twin 1’s score on variable 1 correlated with twin 2’s score on variable 2, which are calculated separately for MZ and DZ twins. The examination of shared variance between traits can be further extended to test the aetiology of the variance that is common between traits and of the residual variance that is specific to individual traits.

It is possible to apply structural equation modelling to decompose latent factors into A, C and E components, applying models such as the common pathway model. The **common pathway model** is a multivariate genetic model in which the variance common to all measures included in the analysis can be reduced to a common latent factor, for which the A, C and E components are estimated. As well as estimating the aetiology of the common latent factor, the model allows for the estimation of the A, C and E components of the residual variance in each measure that is not captured by the latent construct^76^. The common pathway model estimates the extent to which the general factor is explained by A, C and E.

A further multivariate twin method, grounded in SEM is the **Cholesky decomposition**, which allows to examine the genetic and environmental underpinnings of the associations between multiple variables or latent factors. The Cholesky approach, similar to hierarchical regression, parses the genetic and environmental variation in each trait into that which is accounted for by traits that have been previously entered into the model and the variance which is unique to a newly entered trait. This allows, for example, to partition the genetic and environmental variance that is common across cognitive, noncognitive and achievement measures from the genetic and environmental variance that is common between noncognitive skills and achievement, independently of that accounted for by cognitive ability. Cholesky decompositions were conducted on latent dimensions of cognitive (see Supplementary Table 6) and noncognitive (see Supplementary Tables 3 and 4) skills and observed variation in academic achievement.

##### Genetic analyses: Genomic structural equation model (SEM)

Genomic SEM^50^ is an approach to conduct multivariate genome-wide association (GWA) analyses. Based on the principles of SEM widely used in twin analyses and integrated with LD score regression^77^, Genomic SEM jointly analyses GWA summary statistics for multiple traits to test hypotheses about the structure of the genetic covariance between traits. Here we employed Genomic SEM to create latent GWAS summary statistics for unmeasured traits based on other traits for which GWAS summary statistics exist. Recent work applied a GWAS-by-subtraction approach^20^ leveraging GWA studies of educational attainment (EA^17^) and cognitive performance (CP^17,78^) to obtain a GWA of noncognitive skills. The GWA-by-subtraction approach estimates, for each single nucleotide polymorphism (SNP), an effect on EA that is independent of that SNP’s effect on CP (therefore indexing residual *noncognitive* SNP effects). The model regresses the EA and CP summary statistics on two latent variables, *Cog* and *NonCog*. EA and CP are both regressed on the *Cog* latent variable and only EA is regressed on the *NonCog* latent factor. The *Cog* and *NonCog* factors are specified to be uncorrelated and residual covariances are set to zero. *Cog* and *NonCog* are then regressed on each SNP, iterating across all SNPs in the genome.

We extended the GWAS-by-subtraction with the aim of obtaining potentially more fine-grained cognitive and noncognitive factors. Specifically, the model was extended as follows: Loading exclusively on the *Cog* factor: five UK Biobank cognitive traits (Cognitive Performance^78^, Symbol Digit Substitution, Memory, Trail Making Test and Reaction Time)^39^. Loading on both the *Cog* and *Noncog* factors: educational attainment^17^, Townsend deprivation index (http://www.nealelab.is/uk-biobank/), and income^40^. An additional difference from the original GWAS-by-subtraction is that we let residual variances vary freely (i.e., we did not constrain them to 0; see Figure 3A and supplementary Table 14).

##### Genetic analyses: Construction of polygenic scores (PGS) and PGS analyses

Polygenic scores (PGS) were calculated as the weighted sums of each individual’s genotype across all single nucleotides polymorphisms (SNPs), using LDpred weights^79^. LDpred is a bayesian shrinkage method that corrects for local linkage disequilibrium (LD; i.e. correlations between SNPs) using information from a reference panel (we used the target sample (TEDS) limited to unrelated individuals) and a prior for the genetic architecture of the trait. We constructed PGS using an infinitesimal prior, that is assuming that all SNPs are involved in the genetic architecture of the trait, as this has been found to perform well with highly polygenic traits such as educational attainment, and in line with the approach adopted by Demange et al.^20^. In regression analyses, following from Demange et al.^20^, both the Cog and NonCog PGSs were included in multiple regressions together with the following covariates: age, sex, the first 10 principal components of ancestry, and genotyping chip and batch.

##### Genetic analyses: Within and between family analyses

We conducted within-sibling analyses using DZ twins to estimate family-fixed effects of both cog and non-cog PGS on achievement across development^31^. A mixed model was fit to the data including a random intercept to adjust for family clustering, and two family-fixed effects in addition to covariates (age, sex, the first 10 principal components of ancestry, and genotyping chip and batch): a between-family effect indexed by the mean family PGS (i.e. the average of the DZ twins’ PGS within a family), and a within-family effect, indexed by the difference between each twin’s PGS from the family mean PGS.

##### Genetic analyses: Gene x Environment interaction analyses

We conducted gene-environment (GxE) interaction analyses to test whether SES moderated the effects of the cognitive and noncognitive PGS prediction on academic achievement over development. Multiple regression models were fitted including Cog and NonCog PGS, SES and their two-way interactions after adjusting for covariates (as above) and two-way interactions between predictors and covariates. Analyses were repeated with the PGS from Demange et al.^20^, as sensitivity analyses. We adjusted for multiple testing using the Benjamini–Hochberg false discovery rate (FDR) method^74^ for all PGS analyses, at an alpha level of .05.
